# Heart Rate Variability Predicts Therapy Outcome in Anxiety Disorders: The Role of Inhibitory Learning

**DOI:** 10.1007/s10484-025-09686-1

**Published:** 2025-01-20

**Authors:** Sabrina Fagioli, Darcianne K. Watanabe, Julian Koenig, Matthew Free, Russell H. Fazio, Michael W. Vasey, Julian F. Thayer

**Affiliations:** 1 Department of Education, Roma Tre University, Rome, Italy; 2 School of Social Ecology, University of California, Irvine, CA USA; 3 Faculty of Medicine, Department of Child and Adolescent Psychiatry, Psychosomatics and Psychotherapy, University Hospital Cologne, University of Cologne, Cologne, Germany; 4 Columbus OCD and Anxiety Clinic, Columbus, OH USA; 5 Department of Psychology, The Ohio State University, Columbus, OH USA; 6 Department of Psychological Science, University of California, Irvine, CA USA

**Keywords:** Heart rate variability, Vagal activity, Return of fear, Exposure therapy, PIAT, Inhibitory learning

## Abstract

Exposure therapy has been shown to be useful for the treatment of anxiety disorders. However, there are individual differences in the extent to which this intervention is effective in reducing symptoms, and a substantial number of patients may experience a return of fear (ROF). The factors associated with successful therapy outcomes are an important topic of investigation as these factors might influence the nature of the interventions as well as enhance our understanding of the process associated with the disorder and its treatment. Here, we investigated the effect of resting heart rate variability (HRV) on ROF following exposure therapy in social phobics. In particular, using path modeling, we assessed the hypothesis that resting HRV prospectively predicts inhibitory learning, which, in turn, prospectively predicts ROF at follow-up. Forty adult participants (60% female) diagnosed with Social Anxiety Disorder were assigned to a single massed exposure therapy session. Self-reported behavioral and physiological responses were recorded pre-treatment, immediately following treatment, and at one-month follow-up. The Personalized Implicit Association Task (PIAT) was used as an implicit measure of inhibitory learning, and HF-HRV was taken as a measure of vagal activity. Results revealed that those with high pre-treatment HRV reported less negative implicit attitude towards public speaking after exposure (*b* = -0.044, *p* =.047) and showed reduced residual symptoms one month after treatment. (*b* = 2.247, *p =*.013). Taken together these results support exposure therapy models that emphasize the importance of inhibitory learning in extinction and are consistent with research linking HRV to inhibition.

## Introduction

Exposure therapy is one of the most effective treatments for anxiety disorders and specific phobias. Despite the dramatic declines in fear in the context of therapy, treatment gains often degrade over time, and as many as 19–62% of successfully treated patients experience some degree of “return of fear” (ROF) (Boschen et al., 2009; Craske & Mystkowski, 2006; Rachman, 1966, 1989; Rose & McGlynn, 1997). Empirical evidence on ROF’s predictors has yielded contradictory results (Craske et al., 2008a; Rose & McGlynn, 1997). Many variables commonly regarded as predictors of ROF, such as high levels of pre-treatment fear, and fear reduction during the exposure therapy or at the end of the treatment, have not been unambiguously associated with ROF at follow-up (Craske et al., 2008). Therefore, a deeper understanding of ROF’s determinants appears crucial to refine treatment goals and maximize the long-term clinical outcomes from exposure therapy.

It is widely accepted that extinction learning is an integral part of exposure therapy (Bouton, 1993; Craske & Mystkowski, 2006). According to classical conditioning, extinction occurs when an individual learns to decouple the association between a conditioned stimulus (CS) and an unconditioned stimulus (US). In exposure therapy, extinction is accomplished when patients repeatedly confront feared stimuli in the absence of harm, thus learning a new non-threat association (i.e., inhibitory) that competes with the older threat association (Bouton, 1993; Craske et al., 2014a; Jacoby & Abramowitz, 2016; Lang et al., 2006; Myers & Davis, 2007). Deficits in extinction learning, and more specifically in the inhibition learning process, have been suggested to have a role in ROF, and much clinical effort is devoted to optimizing inhibitory learning during exposure to enhance treatment efficacy (Craske et al., 2014a; Craske, Waters, Craske et al., 2008a, b; Lissek et al., 2005). Intriguingly, it is possible to hypothesize that for some patients, reduction in fear across exposure therapy occurs without inhibitory learning. Instead, some patients might merely learn skills that enable them to more effectively regulate their response to a feared stimulus. Such effortful skills would require ample cognitive resources and would be useless when self-regulatory capacity is depleted or when cognitive load is high (Jones et al., 2012). In sum, the predictive power of explicit measures of fear reduction is likely limited by their inability to tap inhibitory learning (Fazio et al., 1986; Hermans et al., 2002).

From a social psychology perspective, phobias can be conceptualized as inappropriately negative and highly accessible attitudes toward a situation or object (Fazio et al., 1986). In particular, two paths can be identified to overcome fear. On the one hand, the attitude object and its affective evaluation can be automatically retrieved from memory without any conscious effort upon the mere presentation of the object. In this case, the behavior or judgment will be influenced by the automatic activation of the attitude, regardless of any motivational concern. On the other hand, the automatically activated fear may be regulated deliberately by effortful, controlled processes involving an interplay of automatic and controlled processes consistent with a dual-process model (Fazio & Olson, 2014). Accordingly, exposure therapy may produce changes in the individual’s attitudinal representation of the feared object (i.e., the automatic component), the individual’s skills at controlling that fear (i.e., the controlled component), or both. Vasey et al. (2012) suggested that if exposure therapy is effective in developing fear-management skills but does not affect the internal affective representation of the fear object, the return of fear after treatment is more likely. Therefore, shifts in automatically activated attitudes (i.e., to less negative) should serve as an index of inhibitory learning. Vasey et al. (2012) tested this hypothesis in the context of a single-session, massed exposure treatment for individuals who feared public speaking. Indeed, participants who adopted a less negative attitude toward public speaking post treatment, as measured by a personalized implicit association test (PIAT), appeared to be insulated from ROF. Those whose fear was reduced by mechanisms that did not entail attitude change (i.e., inhibitory learning) were the most vulnerable to ROF one month later. Consistent with other findings, no explicit measure of change in fear (e.g., self-report, physiological) across treatment predicted ROF at follow-up. Thus, it appears that the PIAT may be a useful index of inhibitory learning and a predictor of ROF.

Critically, although the importance of inhibitory learning is well supported, what enhances inhibitory learning is poorly understood (Craske et al., 2014a; Jacoby & Abramowitz, 2016). Basic neuroscience and neuroimaging studies have significantly advanced our understanding of neurobiological mechanisms underlying extinction learning. These studies’ findings suggest that a network of prefrontal and limbic areas, including the amygdala and the medial prefrontal cortex interact dynamically to regulate neural inhibition during extinction (Delgado et al., 2008; Graham & Milad, 2011; Milad et al., 2014; Myers & Davis, 2007; Phelps et al., 2004; Sotres-Bayon et al., 2006). In particular, while the amygdala has been consistently implicated in the acquisition and expression of conditioned fear (Phelps & LeDoux, 2005), the ventromedial prefrontal cortex (vmPFC) circuitry has been suggested to play a role in the extinction of the conditioned fear through the active modulation of specific regions within the amygdala (Quirk et al., 2003, 2006). Consistently, functional neuroimaging studies in humans showed that increased vmPFC activity attenuated the response of the amygdala to negative emotions (Indovina et al., 2011; Lieberman et al., 2007), supporting the hypothesis that the reduced engagement of vmPFC mechanisms is associated with impoverished neural inhibitory regulation and deficits in inhibitory learning. One study found that individuals who showed greater activation in the vmPFC during early extinction learning had a greater reduction in public speaking anxiety post-exposure (Ball et al., 2017). This highlights the need for therapeutic strategies targeted at enhancing inhibitory regulation through the engagement of vmPFC (Craske et al., 2014b; Indovina et al., 2011).

Interestingly, the capacity of the PFC to exert inhibitory control over the limbic structures may be indexed by resting state vagally mediated heart rate variability (HRV) (Thayer et al., 2009, 2012). Cardiac vagal activity, which represents the contribution of the parasympathetic nervous system to cardiac regulation, has been implicated in the regulation of several physiologic systems, including social, executive, and affective functions (Park & Thayer, 2014; Thayer & Brosschot, 2005; Thayer & Lane, 2000). High vagal activity has been associated with the individual’s general ability to adapt flexibly to a changing environment (Thayer & Lane, 2009) and has been regarded as a protective factor against psychological disease (Jenness et al., 2019). According to the Neurovisceral Integration Model there is a direct connection between the prefrontal cortex and the heart through the central autonomic network and the vagus nerve (Thayer & Lane, 2009). Several neuroimaging studies provided evidence that both the prefrontal cortex and the amygdala are associated with vagal function as indexed by HRV (Thayer et al., 2009, 2012; Sakaki et al., 2016), and that the prefrontal cortex may modulate the activity of subcortical structures via the vagus nerve (Pappens et al., 2014; Wendt et al., 2015; Steinfurth et al., 2018). Accordingly, a recent study showed that extinction of conditioned fear as indexed by the startle blink response, a widely used index of learning processes associated with extinction, was more pronounced in those with high resting HRV compared to those with lower resting HRV (Wendt et al., 2015). Specifically, in the conditioning phase, the participants were presented with pairs of geometrical shapes signaling threat or safety, that is, the presence or absence of a train of unpleasant electrical pulses, respectively. Then, in the test phase, the threat signal was presented together with the safety signal to test for conditioned fear inhibition; or, the threat signal was paired with a novel stimulus to test for external inhibition. Finally, during extinction, the threat signal occurred without the aversive unconditioned stimulus to investigate fear extinction. Results revealed that those with high resting HRV, but not with low HRV, showed significant conditioned fear inhibition as indexed by a reduction in startle potentiation when the threat and the safety signals were presented together. Moreover, high levels of resting HRV, but not low levels of resting HRV, were associated with significant extinction of conditioned startle potentiation (Wendt et al., 2015). Thus, greater resting HRV may be associated with enhanced inhibitory learning.

Given these considerations, we aimed to investigate the predictive power of vagally-mediated HRV on the long-term effects of an exposure-based treatment, as mediated by the strength of inhibitory learning indexed by a PIAT. In particular, we hypothesized that high vagal activity would impact the regulation of neural inhibition, thus facilitating inhibitory learning during exposure. This was expected to reduce the probability of encountering ROF at follow-up, therefore optimizing inhibitory learning during exposure therapy and enhancing the treatment efficacy.

## Method

### Participants

The present study is based on our analysis of data from our previous longitudinal study (Vasey et al., 2012). The sample comprised 40 adult participants (female: 60%; mean age: 22.4 ± 5.7; range: 18–46) who met diagnostic criteria for Social Anxiety Disorder (SAD) in the context of public speaking (with 20% of the participants also meeting criteria for SAD – Generalized Type) according to the DSM-IV-TR (APA, 2000). Exclusion criteria were: (1) Current major depressive disorder or suicidal ideation; (2) Current or past diagnosis of bipolar disorder, schizophrenia, other psychosis, or organic mental syndrome; or (3) Current psychosocial treatment. Participants were medication free, with the exception of three patients who used antidepressant or anxiolytic drugs at a stable dose during the study. The study was approved by the local Ethics Committee and all the participants gave written informed consent before taking part in the study.

### Self-Report Measures

The participants were administered a series of self-report questionnaires, including (1) the Personal Report of Confidence as a Speaker (PRCS), a widely used measure of public speaking anxiety (Paul, 1966); (2) the Brief Fear of Negative Evaluation Scale (BFNE), a measure of fear of social evaluation (Leary, 1983); (3) the State-Trait Anxiety Inventory – State Version (STAI-Y Form), a measure of anxiety at a particular moment (Spielberger et al., 1983); (4) Behaviours Checklist (BCL), a measure of anxiety during a speech (Mansell & Clark, 1999); and (5) Subjective Units of Distress (SUDS), a measure of the intensity of distress (Wolpe, 1969). Cronbach’s alphas across all time points ranged from 0.91 to 0.94 (PRCS), 0.94 to 0.96 (BFNE), 0.92 to 0.97 (STAI-Y), 0.93 to 0.94 (BCL). These instruments are described in further detail in Vasey et al. (2012).

### Behavior Approach Task (BAT)

The Behavioral Approach Task consisted of a 5-min speech to be delivered facing a video camera in the presence of the experimenters. In the follow-up, the participants were also asked to perform a BAT facing a live audience. Independent undergraduate assistant observers rated the overall effectiveness and quality of the speeches (see Vasey et al., 2012 for further details). ICCs for these average ratings ranged from 0.82 to 0.91. ECG was measured continuously during the BAT assessment. The first five minutes of the relaxation period, during which no further stimuli were presented, were used to determine HRV. The intensity of experienced anxiety and panic were assessed immediately after each phase on an 11-point scale ranging from 0 (very poor) to 10 (very high).

### Personalized Implicit Association Task

To measure the individuals’ implicit affective evaluation towards public speaking, the Implicit Association Task was used. It provides a measure of implicit associations between objects/concepts and evaluations. The rationale is that response is faster when closely related attributes share the same response key (Greenwald et al., 1998). For the purpose of this study, a personalized single-category version of the classic IAT (PIAT) was administered before and after the exposure treatment (Karpinski & Steinman, 2006; Olson & Fazio, 2004; Vasey et al., 2012). PIAT score at post-treatment measured the attitude representation of the phobic situation following exposure treatment, hence reflecting the strength of the inhibitory learning process. Participants were presented with 10 blocks of 30 images belonging to three different categories: (1) pleasant images (e.g., an ice cream sundae, a gift box, etc.); (2) unpleasant images (e.g., a spider, burnt toasts, etc.) and (3) public speaking images (e.g., a microphone, an audience, etc.). The task was to categorize each image using one of two response keys. On half of the blocks, one key contained the labels “I like” and “Public Speaking” together, while the other key contained the label “I don’t like.” On the remaining blocks, one key contained the labels “I like,” while the other key contained the label “I don’t like” and “Public Speaking” together. Two blocks were used as practice trials. The two response mappings were counterbalanced across the remaining eight blocks, with the critical comparison involving the difference in latencies for the two response mappings. Higher scores indicate a more positive attitude, as shown by shorter latencies when “I like” and “Public Speaking” were mapped onto the same response key compared to when “I don’t like” and “Public Speaking” were mapped onto the same key. As such, four scores (two mappings for each of the eight blocks) were averaged to compute a total PIAT score. Across the three PIAT tasks, the average Cronbach’s alpha = 0.66.

### Exposure Treatment

After the baseline assessment, the participants underwent a single session exposure treatment according to the model developed by Tsao & Craske (2000). Specifically, the participants completed four exposure trials during which they were given 2-minutes to prepare prior to delivering 5-minute speeches. SUDs ratings were obtained pre-speech and at 1-minute intervals thereafter. All treatment was supervised by a licensed clinical psychologist and treatment sessions were observed either live or via video recording by the one of the authors to monitor treatment fidelity.

### Physiological Recordings

Cardiac activity was acquired using a Polar ambulatory heart-rate monitor attached to a standard chest belt. Data were transmitted to a model RS800 Polar watch. Heart rate data were not available for two participants due to equipment malfunction.

The ECG signal was filtered online with an 8 to 13 Hz bandpass filter, amplified with the factor 2000, and sampled at a rate of 1000 Hz using a Coulbourn V75-04 bioamplifier (Allentown, PA). Then, the ECG signal was down-sampled to 400 Hz, visually inspected and artifact-corrected using ANSLAB (Blechert et al., 2016). Kubios HRV Analysis (Niskanen et al., 2004) was used to determine frequency-domain measures of vagally mediated HRV, that is, high frequency (HF; 0.15–0.40 Hz) and low frequency (LF; 0.04–0.15 Hz) HRV. Absolute power (ms²) of HF-HRV was determined using an autoregressive algorithm as implemented in Kubios HRV Analysis. Prior to all analyses, HF-HRV and LF-HRV were logarithmized (natural log) to account for deviations from normal distribution and unequal sample sizes. Resting HRV (HF-HRV) was taken as a measure of vagal activity (Laborde et al., 2017).

### Procedure

The participants underwent three assessment sessions. In Session 1, they completed a PIAT and were administered the Structured Clinical Interview for DSM-IV (First et al., 1995) to confirm the diagnosis of SAD and exclude any comorbid diagnosis of affective or psychiatric disorders. In Session 2 (typically within 2-weeks of Session 1), those that met eligibility requirements underwent (i) administration of self-report measures of anxiety (i.e., PRCS, BFNE, STAI); (ii) pre-treatment BAT; (iii) administration of BCL; (iv) exposure treatment; (v) administration of self-report measures of anxiety (i.e., PRCS, BFNE, STAI); (vi) post-treatment BAT; (vii) administration of BCL; (viii) performance on PIAT. In Session 3 (1-month later), participants underwent: (i) performance on a PIAT; (ii) administration of self-report measures of anxiety (i.e., PRCS, BFNE, STAI); (iii) BAT facing a video camera (BAT 1); (iv) administration of BCL; (v) administration of STAI; (vi) BAT facing a live audience (BAT 2); (vii) administration of BCL. Heart rate was recorded continuously during each BAT. Moreover, during each BAT, participants provided two SUDS ratings immediately before and at 1-minute intervals (anticipatory and maximum SUDS, respectively). The clinical assessment was administered by trained doctoral students in Clinical Psychology. Data were lost for two participants at pre- and post-BAT. The procedure is described in greater detail elsewhere (Vasey et al., 2012).

### Statistical Analysis

To investigate whether resting vagal activity has an influence on the regulation of neural inhibition, hence facilitating inhibitory learning during exposure and decreasing the risk of return of fear after 1 month from the treatment path modeling was conducted.

**Fig. 1:**
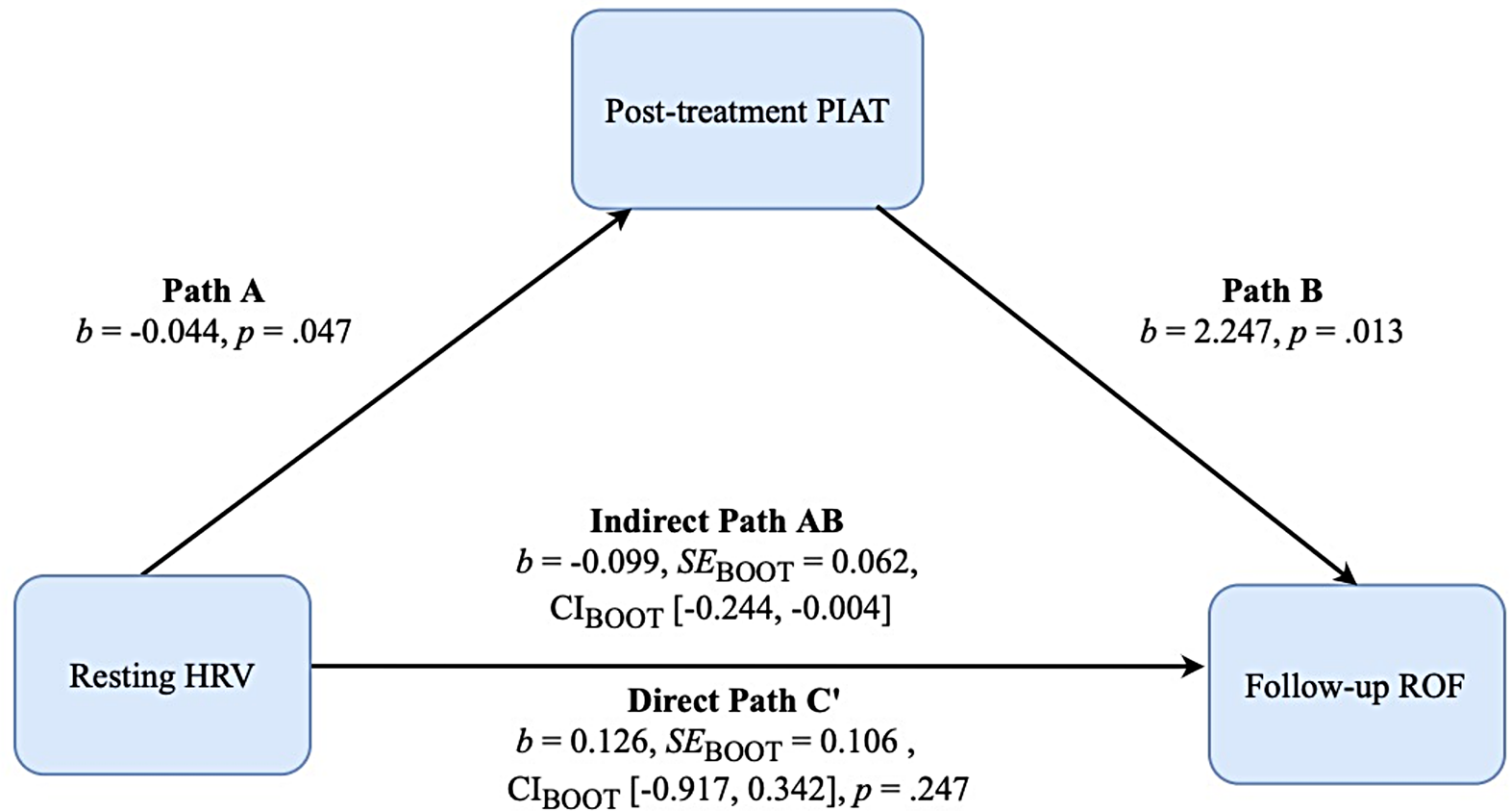
Mediation of resting HRV by post-treatment PIAT on return of fear at 1-month follow-up. *Note* Bootstrap sample size = 20,000. Pre-treatment resting high-frequency heart rate variability (resting HRV). Follow-up return of fear (ROF) measured 1 month post-treatment. Covariates included in the model: pre-treatment personalized IAT (PIAT) scores, aggregated and averaged composite z-scores of state anxiety ratings before and during the speech behavioral approach task at pre- and post-treatment, and low-frequency HRV. *b* = unstandardized regression coefficient; *SE* = standard error; SE_BOOT_ = bootstrapped SE; CI_BOOT_ = bias-corrected bootstrapped 95% confidence intervals. Model summary for dependent variable model: *R*^2^ = 0.85, *F* (6,29) = 27.573, *p* <.001

We assessed the indirect effect of resting-state HRV (predictor), on ROF at follow-up (criterion), considering the level of negative attitude towards public speaking (i.e., post-treatment PIAT score; mediator). ROF was assessed using self-report observer ratings and physiological responses (i.e., heart rate). The self-report measures included the STAI-S, pre-BAT SUDS rating, mean SUDS during BAT, and Behaviors Checklist post-BAT. Observer ratings included nervous appearance during the BAT, and the physiological response was the mean heart rate during the BAT. We computed a measure of ROF for each of these measures, standardized them, and averaged them to compute a composite ROF index for the pre-treatment (ROFCompositePre; *Mz*: 0.005[0.74]), post-treatment (ROFCompositePost; *Mz*: 0.001[0.71]), and follow-up (ROFCompositeFu; *Mz*: 0.010[0.82]) assessments, respectively. The composite index allowed us to capture a more valid index of the treatment gain using a single measure instead of several separate measures, thus maximizing statistical power while reducing the risk of incurring a Type-I error. The three composite scores showed a high internal consistency, as reflected by the Cronbach’s alpha coefficients: ROFCompositePre = 0.85; ROFCompositePost = 0.82; ROFCompositeFu = 0.88, respectively. We assessed the direct effect of resting HRV on ROF at follow-up (i.e., the total effect: HF-HRV on ROFCompositeFu, see Path C, Fig. 1) and the indirect effect mediated by the level of implicit attitude towards public speaking after exposure treatment (i.e., the relationship between HF-HRV and ROF at follow-up via the mediator, post-treatment PIAT score, Path C’). Model covariates included the effect of pre-treatment PIAT score, LF-HRV, and ROFCompositePre and ROFCompositePost scores. We implemented Model 4 of the PROCESS custom dialog for SPSS (Hayes, 2013) to test this mediation Bootstrapped 95% confidence intervals (based on 20,000 bootstrap samples) were used to determine the statistical significance of the direct and indirect effects (Banjanovic & Osborne, 2016; DiCiccio & Efron, 1996).

### Preliminary Analysis

As previously reported (Vasey et al., 2012), paired t-tests showed significant improvements from pre- to post-treatment on all measures (Table 1). Pre-treatment HF-HRV and LF-HRV means (SD) were 6.77 (0.97) and 6.92 (0.78), respectively. A significant reduction in PIAT scores indicated a less negative attitude towards public speaking following treatment. Change on the PIAT was not significantly predicted by change on any other measure following treatment (rs ranged from 0.11 to 0.14, ps > 0.39). On average, post-treatment to follow-up gains persisted for PIAT and observer ratings of speech quality, and further improvement was seen in BFNE scores. In contrast, on average, anticipatory STAI scores for BAT 2 but not BAT 1 increased significantly from post-treatment to follow-up. Pre-speech SUDs ratings trended toward a similar pattern. On average, ROF was observed during BAT 2 but not BAT 1 for maximum SUDs rating. Similarly, during both BATs, ROF was observed for mean heart rate.

**Table 1:** Means and (SDs) of outcome measures at pre-treatment, post-treatment, and follow-up

Measure	Pre-treatment		Post-treatment		Follow-up			
	Mean	(SD)	Mean	(SD)	Mean	(SD)		
Personalized IAT	46.02_a_	(73.13)	-2.59_b_	(58.02)	0.20_b_	(52.22)		
PRCS	23.27_a_	(5.78)	19.62_b_	(7.65)	20.42_b_	(7.64)		
BFNE	41.72_a_	(11.57)	40.10_a_	(11.32)	37.82_b_	(12.22)		

Measure	Pre-treatment		Post-treatment		Speech to camera		Speech to audience	
	Mean	(SD)	Mean	(SD)	Mean	(SD)	Mean	(SD)
Pre-BAT STAI-State Anxiety	53.05_a_	(12.44)	44.32_b_	(9.56)	45.05_b_	(13.35)	47.80_c_	(14.29)
SUDs - anticipatory	50.22_a_	(21.63)	40.21_b_	(17.76)	39.68_b_	(23.16)	$\:{41.18}_{\text{b}}^{1}$	(22.25)
SUDs - maximum	62.05_a_	(23.66)	41.20_b_	(19.04)	43.18_b_	(24.16)	45.92_c_	(21.61)
BCL total	83.30_a_	(26.78)	58.64_b_	(23.38)	$\:{63.65}_{\text{b}}^{2}$	(27.71)	61.25_b_	(27.04)
Mean heart rate during BAT	$\:{91.05}_{\text{a}}^{3}$	(13.56)	$\:{87.79}_{\text{b}}^{3}$	(11.60)	$\:{92.53}_{\text{a}}^{4}$	(14.88)	$\:{91.60}_{\text{a}}^{4}$	(15.31)
Observer rating of speech quality	5.24_a_	(1.61)	5.88_b_	(1.27)	5.84_b_	(1.38)	6.05_b_	(1.28)

*Note*: High-frequency (HF) and low-frequency (LF) heart-rate variability (HRV), Implicit Association Test (IAT), Personal Report of Confidence as a Speaker (PRCS), Brief Fear of Negative Evaluation Scale (BFNE), behavioral approach task (BAT), State-Trait Anxiety Inventory State Version (STAI [Form Y]), Subjective Units of Distress (SUDS), Behaviours Checklist (BCL). Means with different subscripts indicate differences at *p* <.05 (e.g., means denoted with subscript a differ from those with subscript b). However, the two speeches at follow-up were not compared to one another. *N* = 40 except where noted. ^1^Difference from post-treatment: *p* =.062; ^2^Difference from post-treatment: *p* =.073; ^3^*N* = 38; ^4^*N* = 36

### Relation of Resting HF-HRV and ROF to Changes in Inhibitory Learning

Results from bootstrapping revealed a significant indirect effect of resting HRV on ROF at follow-up (b = − 0.0993, SE_BOOT_ = 0.612, CI_BOOT_ [-0.240, − 0.002]. Specifically, for Path-A, higher HRV predicted lower PIAT score after exposure treatment (b = − 0.044, SE = 0.021, 95%CI [-0.088, − 0.001], *p =*.047), subsequently leading to greater reduction of ROF at follow-up (Path-B) (*b* = 2.247, SE = 0.849, 95%CI [0.511, 3.983], *p =*.013) (see Fig. 1). Most notably, once the mediator (i.e., post-treatment PIAT) was entered into the model, the total effect of resting HRV on ROF at follow-up was no longer significant (b = 0.262, SE_BOOT_ = 0.109, CI_BOOT_ [-0.196, 0.249], *p =*.812), indicating mediation by PIAT score after exposure treatment (Baron & Kenny, 1986). No other variables had a significant impact on the model.

Hence, the association between resting HRV and ROF at follow-up was mediated by the implicit attitude toward public speaking. In other words, higher HRV at rest predicted changes in inhibitory learning as assessed by the PIAT, which in turn predicted return of fear 1 month after the exposure treatment.

## Discussion

In this study, we investigated the effect of resting vagally mediated heart rate variability on ROF following a single massed exposure therapy in social phobics. In particular, we tested the hypothesis that higher vagal activity, indexed by pre-treatment resting high-frequency heart rate variability, facilitates extinction learning during exposure through enhancement of inhibitory learning (Thayer et al., 2012; Thayer & Lane, 2009; Wendt et al., 2015). We found that high resting HRV was positively associated with lower subjective distress at one-month follow-up. Crucially, the mediation analysis revealed that high pre-treatment HRV predicted less negative affective representation of the fear object after exposure, which led to decreased residual symptoms at follow-up. Therefore, the association between HRV and ROF was mediated by a modulatory effect of HRV on inhibitory learning processes.

The observed association between high pre-treatment resting HRV and reduced subjective distress is consistent with previous studies showing that heart rate variability before treatment is associated with treatment drop-out and residual symptoms after treatment such that higher HRV at pre-treatment is associated with better treatment outcomes (Wendt et al., 2018). More generally, having high HRV has been systematically associated with higher emotional well-being (Mather & Thayer, 2018), being linked with lower levels of worry and rumination (Ottaviani et al., 2016), lower anxiety (Chalmers et al., 2014), and better emotional-(Appelhans & Luecken, 2006) and self-regulation (Holzman & Bridgett 2017). In contrast, low resting HRV has been associated with increased risk of depression (Kemp et al., 2010), anxiety disorders (Chalmers et al., 2014), and cardiovascular disease (Thayer et al., 2010), among other health outcomes, and has been therefore regarded as a transdiagnostic biomarker of mental illness (Beauchaine & Thayer, 2015). Thus, substantial evidence exists to support the notion that decreased HRV can be viewed as a predictor of psychopathology (Beauchaine & Thayer, 2015; Kemp & Quintana, 2013; Thayer & Brosschot, 2005). Findings from our study confirmed and extended these results, providing insights into the modulatory role of resting HRV on inhibitory learning.

In the present experiment, resting HF-HRV prior to treatment predicted inhibitory learning, and this, in turn, predicted ROF 1 month after the treatment. First, this result supports the hypothesis that treatment gains are more durable when the automatically activated associations between the fear object and its affective evaluation change (Vasey et al., 2012). This aligns with research conducted on evaluative conditioning showing that even though exposure treatment entails a significant extinction of the association between the predictive CS – US relationship, the CS remains associated with a negative valence (Hermans et al., 2002; Huijding & de Jong, 2009). From a clinical perspective, the patients who, after exposure, learn to control their dysfunctional reaction (e.g., avoidance) towards the fear object (e.g., spiders) and continue to experience a negative affect toward it are more prone to develop ROF (Dirikx et al., 2004; Huijding & de Jong, 2009; Vasey et al., 2012). Accordingly, our findings suggest that helping clients change automatically activated associations may be an important target for intervention.

Second and most importantly, our findings suggest that increasing HRV at rest may be useful for maximizing the efficacy of exposure treatment and reducing residual symptoms at follow-up. In the present experiment, having high levels of pre-treatment resting HRV predicted greater changes in the negative affective association between the CS and the US due to exposure treatment, as measured by the PIAT. Patients who more effectively reduced their negative implicit attitude towards public speaking were less likely to experience ROF one month after treatment. In particular, our finding that high pre-treatment resting HRV predicts less spontaneous return of fear by enhancing extinction learning, and more generally, inhibitory processing during exposure, is in line with theoretical models suggesting a common inhibitory cortico-subcortical neural circuit that regulates psychological processes like emotion and cognition, and health-related physiological processes, and that can be indexed with HRV (Thayer & Lane, 2000, 2009). As highlighted in the introduction, extinction learning has been suggested to be regulated by the tonic inhibitory control of the vmPFC over the amygdala (Indovina et al., 2011; Wendt et al., 2015; Ball et al., 2017), which is responsible for the stress response (Motzkin et al., 2015). Indeed, the activation or disinhibition of the central nucleus of the amygdala (CeA), which is the major efferent source of modulation of cardiovascular, autonomic, and endocrine responses, leads to a modulation of heart rate and heart rate variability (Thayer & Lane, 2009). Intriguingly, the importance of neural inhibitory regulation in preventing stress-related diseases has been stressed in the Generalized Unsafety Theory of Stress (GUTS; (Brosschot et al., 2017). According to this theoretical model, the stress response is a default response that is normally under tonic inhibitory control, rather than a response to threat (Brosschot et al., 2018). When safety is perceived, there is PFC inhibition of subcortical areas, including the amygdala, and this is reflected by higher resting HRV. Instead, if a generalized perception of unsafety is detected, PFC inhibition is withdrawn, and the default stress response is unleashed, as indexed by lower resting HRV (Brosschot et al., 2017, 2018).

Taken together, these studies suggest that vagally mediated heart rate variability may be a reliable indicator of prefrontal inhibitory capability (Thayer et al., 2012) and, more generally, of the strength of inhibitory learning during exposure therapy (Pappens et al., 2014; Wendt et al., 2015). Accordingly, our findings showed that HRV is a good predictor of treatment outcome, such that having high pre-treatment HRV predicted lower ROF one month after treatment. Although future research is necessary to determine if treatment gains persist at a later point in time after treatment, interventions aimed at increasing pre-treatment HRV may have a meaningful effect on improving inhibitory learning, treatment outcomes, and mental health. For instance, voluntary slow breathing and HRV biofeedback have been meta-analytically shown to increase HRV and improve anxiety (Goessl et al., 2017; Laborde et al., 2022; Lehrer et al., 2020) and in clinical trials (Jung et al., 2024; Yoo et al., 2022).

### Limitations

These results should be considered in light of several limitations. First, whereas participants met the DSM-IV criteria for SAD, only around 20% of our sample met the criteria for generalized SAD. Thus, a single massed treatment session may not produce the same ROF results in a group with more severe SAD. However, we do not believe this would influence the reliability of HRV as a predictor of prefrontal inhibitory learning or treatment outcome following exposure therapy (Wendt et al., 2015, 2018). Second, we could not determine whether certain higher-order cognitive processes (e.g., perceived control over harm expectancy) contributed to reduced ROF at follow-up (Hofmann et al., 2008), which could be tested in future studies. Finally, caution should be taken when generalizing to broader populations, as our sample size was relatively small.

In summary, these results support models of exposure therapy that emphasize the importance of inhibitory learning in extinction and are consistent with research linking HRV to inhibition. Importantly, results expand existing knowledge about factors that may enhance inhibitory learning during exposure therapy and suggest that higher pre-treatment resting HRV may both optimize prefrontal inhibitory learning and enhance exposure treatment efficacy. Future research in larger samples may provide further clarification of our findings.

## Data Availability

The data are available from the authors upon reasonable request.
